# Increasing the risk competence of patients: an interdisciplinary task

**DOI:** 10.3205/000216

**Published:** 2015-07-09

**Authors:** Harald Schweim

**Affiliations:** 1 Drug Regulatory Affairs, Rheinische Friedrich-Wilhelm-University, Bonn, Germany

## Editorial

The public debate on health risks has strongly increased in Germany in recent years. One reason for this increase may be the often dramatic manner of news reporting in the media that informs the public on current outbreaks of BSE, bird flu or Ebola. Afterwards, it often became apparent that the sometimes fierce discussions had not addressed the core of the problem at all, and the public was left with feelings of insecurity.

In this situation, the Committee of Research into Natural Medicines (Komitee Forschung Naturmedizin – KFN) decided to ask an interdisciplinary working group what could be done to increase the risk competence of interested lay people in this respect.

The working group chaired by Michael Koller comprises:

- Educational research: Professor Ulrich Hoffrage, University of Lausanne, Switzerland
- Medicine: Professor Michael Koller, Centre for Clinical Studies, University of Regensburg, Germany
- Health research: Professor Ingrid Mühlhauser, Institute for Health Sciences of the University of Hamburg, Germany
- Pharmacology: Professor Harald Schweim, Department of Drug Regulatory Affairs of the University of Bonn, Germany
- Toxicology: Professor Ralf Stahlmann, University Hospital Charité Berlin, Germany
- Cultural sciences: Professor Michael Wolffsohn, University of the German Armed Forces, Munich, Germany

After short key statements and a discussion at a seminar held in Munich on January 24^th^ and 25^th^, 2014, relevant aspects were compiled, grouped and assigned to the individual participants for further elaboration by means of the nominal group technique. The results of this process constitute the content of this multipart publication.

Ingrid Mühlhauser and her team mainly focus on the communication between physicians and patients [1]. Based on their long experience in evidence-based medicine, this team strongly believes that published scientific data should also be continuously integrated into the communication between physicians and laypeople.

Ralf Stahlmann states that toxicological findings can only be correctly interpreted if the physiology and pathophysiology of animals and humans is well understood [2]. He emphasises that experimental results or concentrations of poisonous substances in the human environment are often taken out of their research context in order to satisfy the popular media’s craving for sensations.

Harald Schweim concentrates on public communication and media [3]. He emphasises the role of the media in the public reception of health issues and shows the areas that are particularly often affected by problems and the reasons for such problems. One important aspect is health education, which is not only communicated in the framework of school education but also as part of adult education, thus health education is accompanying people for the rest of their lives.

The paternalistic decision model in medicine has been increasingly replaced by the emancipation of patients. Simultaneously, the internet provides unfiltered information on just any topic. Michael Koller analyses the fact that medical decisions are nowadays made not only according to medical aspects but also according to social aspects and considers the resulting consequences [4].

Ulrich Hoffrage particularly investigates disruptive factors that often occur in the communication between physicians and patients [5]. Hoffrage shows areas in which misconceptions about risk factors lead to the inflation of certain risks of individual patients or to the overestimation of the success of preventive measures. Simultaneously, he demonstrates how such problems may be avoided solely by the mode of statistical presentation.

Michael Wolffsohn concentrates on the social perspective of risk competence and – deduced from general historical empiricism – addresses the question of the willingness of today’s ‘post heroic’ society to take risks [6]. He also investigates if patient emancipation represents a real opportunity or if it is just an illusion that is kept alive by society out of economic necessity.

The working group will further analyse this subject and investigate if patient emancipation has really decreased individual risk. However, the ethical question if decreasing physician responsibility may be accompanied by increasing patient responsibility remains unanswered.
